# Supplementary material for: Sleep restriction impairs visually and memory-guided force control
Source: PLoS One. 2022 Sep 2;17(9):e0274121. doi: 10.1371/journal.pone.0274121 (PMC9439228; doi:10.1371/journal.pone.0274121)
Supplement: S2 Table — Note. Values given in Estimate (Standard Error); VG = Visually Guided; MG = Memory-Guided; TST = Total Sleep Time; KSS = Karolinska Sleepiness Scale. Second series of random intercept models with potential covariates. The best-fitting model of the series—determined by AIC—was Model 4: KSS. ***p < 0.001; ** p < 0.01; *p < 0.05. (DOCX) [file pone.0274121.s002.docx]

**Supplementary Table 2. Random intercept models with potential covariates**

|  | **Model 1:**  **Race** | **Model 2:**  **Age** | **Model 3:**  **TST** | **Model 4:**  **KSS** | **Model 5:**  **All covariates** | **Model 6**  **TST and KSS** |
| --- | --- | --- | --- | --- | --- | --- |
| Intercept (Day=Baseline, Vision=VG, KSS=4, TST=8,  Age=22) | 24.80  (0.20)*** | 24.78  (0.15)*** | 24.70  (0.14)*** | 24.74  (0.17)*** | 24.70  (0.26)*** | 24.70  (0.17)*** |
| Day (Restriction vs. Baseline) | -0.64  (0.02)*** | -0.64  (0.02)*** | -0.22  (0.07)** | -0.34  (0.02)*** | -0.12  (0.07) | -0.12  (0.07) |
| Day (Recovery vs. Baseline) | 0.18  (0.02)*** | 0.18  (0.02)*** | 0.16  (0.02)*** | 0.20  (0.02)*** | 0.19  (0.02)*** | 0.19  (0.02)*** |
| Vision (MG vs. VG) | -0.57  (0.02)*** | -0.57  (0.02)*** | -0.57  (0.02)*** | -0.57  (0.02)*** | -0.57  (0.02)*** | -0.57  (0.02)*** |
| Day (Restriction vs. Baseline) x Vision (MG vs. VG) | 0.19  (0.02)*** | 0.19  (0.02)*** | 0.19  (0.02)*** | 0.19  (0.02)*** | 0.19  (0.02)*** | 0.19  (0.02)*** |
| Day (Recovery vs. Baseline) x Vision (MG vs. VG) | -0.13  (0.02)*** | -0.13  (0.02)*** | -0.13  (0.02)*** | -0.13  (0.02)*** | -0.13  (0.02)*** | -0.13  (0.02)*** |
| Race (Asian) | 0.05  (0.38) |  |  |  | 0.23  (0.48) |  |
| Race (Black) | -0.18  (0.38) |  |  |  | -0.28  (0.48) |  |
| Age |  | -0.01  (0.05) |  |  | 0.02  (0.07) |  |
| TST |  |  | 0.10  (0.02)*** |  | 0.05  (0.02)** | 0.05  (0.02)** |
| KSS |  |  |  | -0.12  (0.01)*** | -0.12  (0.01)*** | -0.12  (0.01)*** |
| AIC | 1085160.06 | 1085161.94 | 1085129.31 | 1084560.00 | 1084566.80 | 1084558.57 |
| BIC | 1085263.95 | 1085255.43 | 1085222.81 | 1084653.49 | 1084701.85 | 1084662.45 |
| Log Likelihood | -542570.03 | -542571.97 | -542555.66 | -542271.00 | -542270.40 | -542269.29 |
| Num. obs. | 239985 | 239985 | 239985 | 239985 | 239985 | 239985 |
| Num. groups: Participant | 14 | 14 | 14 | 14 | 14 | 14 |
| Var: Participant (Intercept) | 0.31 | 0.29 | 0.28 | 0.41 | 0.50 | 0.41 |
| Var: Residual | 5.38 | 5.38 | 5.38 | 5.37 | 5.37 | 5.37 |

*Note. Values given in Estimate (Standard Error); VG = Visually Guided; MG = Memory-Guided; TST = Total Sleep Time; KSS = Karolinska Sleepiness Scale. Second series of random intercept models with potential covariates. The best-fitting model of the series — determined by AIC — was Model 4: KSS. ***p < 0.001; ** p < 0.01; *p < 0.05.*
