# Supplementary material for: Sleep restriction impairs visually and memory-guided force control
Source: PLoS One. 2022 Sep 2;17(9):e0274121. doi: 10.1371/journal.pone.0274121 (PMC9439228; doi:10.1371/journal.pone.0274121)
Supplement: S3 Table — Note. Values given in Estimate (Standard Error); VG = Visually Guided; MG = Memory-Guided; KSS = Karolinska Sleepiness Scale. Third series of random intercept models with interactions between study design variables and covariates that improved model fit by AIC. The best-fitting model of the series—determined by AIC—was Model 2: Main effects, all interactions. ***p < 0.001; ** p < 0.01; *p < 0.05. (DOCX) [file pone.0274121.s003.docx]

**Supplementary Table 3. Random intercept models with study design and covariates**

|  | **Model 1:**  **Main effects, 2-way interactions** | **Model 2:**  **Main effects, all interactions** |
| --- | --- | --- |
| Intercept (Day=Baseline, Vision=VG, KSS=4) | 24.70 (0.17)*** | 24.73 (0.17)*** |
| Day (Restriction vs. Baseline) | -0.14 (0.02)*** | 0.08 (0.02)*** |
| Day (Recovery vs. Baseline) | 0.22 (0.02)*** | 0.19 (0.02)*** |
| Vision (MG vs. VG) | -0.51 (0.02)*** | -0.56 (0.02)*** |
| KSS | -0.29 (0.01)*** | -0.18 (0.01)*** |
| Day (Restriction vs. Baseline) x Vision (MG vs. VG) | -0.34 (0.03)*** | -0.77 (0.03)*** |
| Day (Recovery vs. Baseline) x Vision (MG vs. VG) | -0.15 (0.02)*** | -0.10 (0.02)*** |
| Day (Restriction vs. Baseline) x KSS | 0.08 (0.01)*** | -0.14 (0.01)*** |
| Day (Recovery vs. Baseline) x KSS | 0.08 (0.01)*** | -0.03 (0.01)*** |
| Vision (MG vs. VG) x KSS | 0.23 (<0.01)*** | 0.02 (0.01)* |
| Day (Restriction vs. Baseline) x Vision (MG vs. VG) x KSS |  | 0.44 (0.01)*** |
| Day (Recovery vs. Baseline) x Vision (MG vs. VG) x KSS |  | 0.23 (0.01)*** |
| AIC | 1081975.85 | 1080386.67 |
| BIC | 1082100.51 | 1080532.11 |
| Log Likelihood | -540975.92 | -540179.34 |
| Num. obs. | 239985 | 239985 |
| Num. groups: Participant | 14 | 14 |
| Var: Participant (Intercept) | 0.41 | 0.41 |
| Var: Residual | 5.31 | 5.28 |

*Note. Values given in Estimate (Standard Error); VG = Visually Guided; MG = Memory-Guided; KSS = Karolinska Sleepiness Scale. Third series of random intercept models with interactions between study design variables and covariates that improved model fit by AIC. The best-fitting model of the series — determined by AIC — was Model 2: Main effects, all interactions. ***p < 0.001; ** p < 0.01; *p < 0.05.*
