# Supplementary material for: Sleep restriction impairs visually and memory-guided force control
Source: PLoS One. 2022 Sep 2;17(9):e0274121. doi: 10.1371/journal.pone.0274121 (PMC9439228; doi:10.1371/journal.pone.0274121)
Supplement: S4 Table — Note. Values given in Estimate (Standard Error); VG = Visually Guided; MG = Memory-Guided; KSS = Karolinska Sleepiness Scale. Fourth series of models, comparing random slopes for day and vision and no random slopes. The best-fitting model of the series—determined by AIC—was Model 4: Random Slopes on Day and Vision. ***p < 0.001; ** p < 0.01; *p < 0.05. (DOCX) [file pone.0274121.s004.docx]

**Supplementary Table 4. Random slopes models with study design and KSS**

|  | **Model 1:**  **Random Intercepts** | **Model 2:**  **Random Slope on Day** | **Model 3:**  **Random Slope on Vision** | **Model 4:**  **Random Slopes on Day and Vision** |
| --- | --- | --- | --- | --- |
| Intercept (Day=Baseline, Vision=VG, KSS=4) | 24.73  (0.17)*** | 24.80  (0.18)*** | 24.80  (0.13)*** | 24.80  (0.16)*** |
| Day (Restriction vs. Baseline) | 0.08  (0.02)*** | -0.34  (0.40) | -0.36  (0.03)*** | -0.84  (0.38)* |
| Day (Recovery vs. Baseline) | 0.19  (0.02)*** | 0.15  (0.24) | 0.13  (0.02)*** | 0.15  (0.23) |
| Vision (MG vs. VG) | -0.56  (0.02)*** | -0.56  (0.02)*** | -0.71  (0.29)* | -0.71  (0.29)* |
| KSS | -0.18  (0.01)*** | -0.01  (0.08) | -0.07  (0.01)*** | -0.02  (0.06) |
| Day (Restriction vs. Baseline) x Vision (MG vs. VG) | -0.77  (0.03)*** | -0.77  (0.03)*** | 0.10  (0.04)** | 0.10  (0.03)** |
| Day (Recovery vs. Baseline) x Vision (MG vs. VG) | -0.10  (0.02)*** | -0.10  (0.02)*** | 0.03  (0.02) | 0.03  (0.02) |
| Day (Restriction vs. Baseline) x KSS | -0.14  (0.01)*** | -0.14  (0.14) | -0.07  (0.01)*** | 0.12  (0.12) |
| Day (Recovery vs. Baseline) x KSS | -0.03  (0.01)*** | -0.04  (0.12) | -0.07  (0.01)*** | 0.02  (0.11) |
| Vision (MG vs. VG) x KSS | 0.02  (0.01)* | 0.02  (0.01)* | -0.21  (0.01)*** | -0.21  (0.01)*** |
| Day (Restriction vs. Baseline) x Vision (MG vs. VG) x KSS | 0.44  (0.01)*** | 0.44  (0.01)*** | 0.31  (0.01)*** | 0.31  (0.01)*** |
| Day (Recovery vs. Baseline) x Vision (MG vs. VG) x KSS | 0.23  (0.01)*** | 0.23  (0.01)*** | 0.31  (0.01)*** | 0.31  (0.01)*** |
| AIC | 1080386.67 | 1068674.43 | 1071257.69 | 1059064.60 |
| BIC | 1080532.11 | 1068871.81 | 1071423.90 | 1059303.54 |
| Log Likelihood | -540179.34 | -534318.21 | -535612.85 | -529509.30 |
| Num. obs. | 239985 | 239985 | 239985 | 239985 |
| Num. groups: Participant | 14 | 14 | 14 | 14 |
| Var: Participant  (Intercept) | 0.41 | 0.42 | 0.24 | 0.33 |
| Var: Participant  Day (Restriction) |  | 1.06 |  | 1.03 |
| Var: Participant  Day (Recovery) |  | 0.74 |  | 0.73 |
| Cov: Participant  (Intercept) x Day (Restriction) |  | -0.27 |  | -0.03 |
| Cov: Participant  (Intercept) x Day (Recovery) |  | -0.35 |  | -0.42 |
| Cov: Participant  Day (Restriction) x Day (Recovery) |  | 0.29 |  | 0.23 |
| Var: Participant  Vision (MG) |  |  | 1.14 | 1.14 |
| Cov: Participant  (Intercept) x Vision (MG) |  |  | -0.12 | -0.08 |
| Cov: Participant  Day (Restriction) x Vision (MG) |  |  |  | -0.58 |
| Cov: Participant  Day (Recovery) x Vision (MG) |  |  |  | 0.01 |
| Var: Residual | 5.28 | 5.02 | 5.08 | 4.82 |

*Note. Values given in Estimate (Standard Error); VG = Visually Guided; MG = Memory-Guided; KSS = Karolinska Sleepiness Scale. Fourth series of models, comparing random slopes for day and vision and no random slopes. The best-fitting model of the series — determined by AIC — was Model 4: Random Slopes on Day and Vision. ***p < 0.001; ** p < 0.01; *p < 0.05.*
