# Supplementary material for: Sleep restriction impairs visually and memory-guided force control
Source: PLoS One. 2022 Sep 2;17(9):e0274121. doi: 10.1371/journal.pone.0274121 (PMC9439228; doi:10.1371/journal.pone.0274121)
Supplement: Supplementary file 4 [file pone.0274121.s001.doc]

file106032d19e0.knit



**Supplementary Table 1. Random intercept models**

|  |  |  |  |  |
| --- | --- | --- | --- | --- |
|  | **Model 1:**  **Intercept-only** | **Model 2:**  **MVC** | **Model 3:**  **Day and Vision M.E.** | **Model 4:**  **Day\*Vision interactions** |
| Intercept (Day=Baseline, Vision=VG) | 24.36 (0.14)\*\*\* | 24.36 (0.14)\*\*\* | 24.77 (0.14)\*\*\* | 24.77 (0.14)\*\*\* |
| Day (Restriction vs. Baseline) |  |  | -0.54 (0.01)\*\*\* | -0.64 (0.02)\*\*\* |
| Day (Recovery vs. Baseline) |  |  | 0.11 (0.01)\*\*\* | 0.18 (0.02)\*\*\* |
| Vision (MG vs. VG) |  |  | -0.55 (0.01)\*\*\* | -0.57 (0.02)\*\*\* |
| Day (Restriction vs. Baseline) x Vision (MG vs. VG) |  |  |  | 0.19 (0.02)\*\*\* |
| Day (Recovery vs. Baseline) x Vision (MG vs. VG) |  |  |  | -0.13 (0.02)\*\*\* |
| AIC | 1092095.50 | 1092095.50 | 1085338.40 | 1085155.91 |
| BIC | 1092126.67 | 1092126.67 | 1085400.73 | 1085239.02 |
| Log Likelihood | -546044.75 | -546044.75 | -542663.20 | -542569.95 |
| Num. obs. | 239985 | 239985 | 239985 | 239985 |
| Num. groups: Participant | 14 | 14 | 14 | 14 |
| Var: Participant (Intercept) | 0.28 | 0.28 | 0.27 | 0.27 |
| Var: Residual | 5.54 | 5.54 | 5.39 | 5.38 |

*Note.
Values given in Estimate (Standard Error); VG = Visually Guided; MG =
Memory-Guided.* *First series of random intercept
models e**stimated
to understand the effects of the fixed design factors of the study on percent
of a participant's MVC during the last 12 sec of each trial.* *The best-fitting model of the series — determined by AIC* *— was Model 4: Day\*Vision
interactions. \*\*\*p < 0.001; \*\* p < 0.01; \*p < 0.05.*
