# Supplementary material for: Sleep restriction impairs visually and memory-guided force control
Source: PLoS One. 2022 Sep 2;17(9):e0274121. doi: 10.1371/journal.pone.0274121 (PMC9439228; doi:10.1371/journal.pone.0274121)
Supplement: Attachment — Submitted filename: comments_R1.docx [file pone.0274121.s007.docx]

The authors responded to most of the comments raised by the two reviewers well, but it still needs details and clarification about how they performed the statistical analysis and reported the results. The authors should provide more details about how the force data were analyzed to perform the statistical analysis.

**Methods**

#179-181: The authors did not fully explain the normalization procedure using the MVC values and how the force data were analyzed.

#183: Please provide readers with details for the levels of each factor sooner than later (e.g., two levels, no visual feedback, and visual feedback). Readers need to wait until seeing that information in lines 203~204.

#187: Please provide readers with details for what variables were used as dependent variables in the analysis (e.g., How was the performance of motor force production evaluated? mean of force data normalized by MVC values for each day?)

**Results**

#234: Please provide a quantitative information that explains the model selection (e.g., AIC values).

\# Table 2. The authors stated that there are two factors in the Method, but there seemed to be another factor (KSS; e.g., #282). It is confusing which one is right. Could the authors clarify this issue?

**Discussion**

#402-403: Please provide references to support that the force level used in the current study matches the force levels required for performing some activities of daily living.
