# Supplementary material for: Sleep restriction impairs visually and memory-guided force control
Source: PLoS One. 2022 Sep 2;17(9):e0274121. doi: 10.1371/journal.pone.0274121 (PMC9439228; doi:10.1371/journal.pone.0274121)
Supplement: Attachment — Submitted filename: response to reviewers_revisions2_v2.docx [file pone.0274121.s008.docx]

**REVIEWER #1:**

The authors responded to most of the comments raised by the two reviewers well, but it still needs details and clarification about how they performed the statistical analysis and reported the results. The authors should provide more details about how the force data were analyzed to perform the statistical analysis.

Methods

#179-181: The authors did not fully explain the normalization procedure using the MVC values and how the force data were analyzed.

**Author Response:**
We have updated the end of the “Motor Force task” section to include information about how the force data were normalized to MVC.

Line 181-183: *“Force data were collected in newtons and were divided by the participant’s MVC measured on the same day, multiplied by 100%. Therefore, the data were analyzed as a percent of MVC.”*

#183: Please provide readers with details for the levels of each factor sooner than later (e.g., two levels, no visual feedback, and visual feedback). Readers need to wait until seeing that information in lines 203~204.

**Author Response:**
We named the factor levels earlier in the statistical analysis section, where the reviewer indicated. We also named the explored covariates in the same sentence.

Line 186-189: *“Therefore, the analysis design was repeated measures with two main design factors — day (baseline, sleep restriction, recovery sleep) and vision condition (visually guided and memory-guided) — and four potential covariates (race, age, TST, and KSS score).”*

#187: Please provide readers with details for what variables were used as dependent variables in the analysis (e.g., How was the performance of motor force production evaluated? mean of force data normalized by MVC values for each day?)

**Author Response:**
We provided detailed on the exact dependent measure used in the statistical analyses.

Line 190-193: *“We used a mixed effects multilevel approach to analyze the effects of sleep restriction and visual feedback on mean force produced in the last 12 seconds of each trial, normalized by MVC, in addition to modeling how the effects of day and vision condition varied across individuals.”*

Line 198-200: *“A series of models were estimated in a two-level multilevel framework using the lme4 package [36] in R [37] to model the mean force, normalized by MVC, across days and vision condition, nested within participants.”*

Results

#234: Please provide a quantitative information that explains the model selection (e.g., AIC values).

**Author Response:**
We added AIC fits for the final model (allowing variation due to levels of repeated measures) and for the model that assumed no variation due to repeated measures factors.

Line 186-189: *“The model including random slopes for the day and vision condition (AIC = 1.06x10^6) fit the data better than the model assuming no variation due to day and vision across individuals (AIC = 1.08x10^6).*

In total, 16 models were built-up and consecutively compared for fit to the data. The authors feel that listing all model fits would take space and detract from the results; therefore, all other AIC values (and other model fit values) are provided in supplementary tables.

\# Table 2. The authors stated that there are two factors in the Method, but there seemed to be another factor (KSS; e.g., #282). It is confusing which one is right. Could the authors clarify this issue?

**Author Response:**
While there were two design factors, we also included any covariate that significantly improved the model — KSS score was the only covariate that improved model fit to the data. This has been clarified in manuscript in the following lines.

Line 186-189: *“Therefore, the analysis design was repeated measures with two main design factors — day (baseline, sleep restriction, recovery sleep) and vision condition (visually guided and memory-guided) — and four potential covariates (race, age, TST, and KSS score).”*

Line 244-246: *“The best-fitting model, shown in Table 2, included main effects for day, vision condition, KSS score, and the three-way interaction among day, vision condition, and KSS score.”*

Discussion

#402-403: Please provide references to support that the force level used in the current study matches the force levels required for performing some activities of daily living.

**Author Response:**
This sentence has been revised to be more general, and citations have been added.

Line 402-406: *“Grip force of 20-40% MVC is important for activities of daily living such as writing, unlocking a door, and tying shoes (Marshall & Armstrong, 2004), but it may not be directly applicable to other activities requiring higher or lower grip force (such as operating heavy machinery).”*

**References**

Marshall, M. M., & Armstrong, T. J. (2004). Observational Assessment of Forceful Exertion and the Perceived Force Demands of Daily Activities. *Journal of Occupational Rehabilitation*, *14*(4), 281–294. https://doi.org/10.1023/B:JOOR.0000047430.22740.57
